# The Use of Germinants to Potentiate the Sensitivity of *Bacillus anthracis* Spores to Peracetic Acid

**DOI:** 10.3389/fmicb.2016.00018

**Published:** 2016-01-29

**Authors:** Ozgur Celebi, Fatih Buyuk, Tom Pottage, Ant Crook, Suzanna Hawkey, Callum Cooper, Allan Bennett, Mitat Sahin, Leslie Baillie

**Affiliations:** ^1^Department of Microbiology, Faculty of Veterinary Science, University of KafkasKars, Turkey; ^2^Biosafety Unit, Public Health EnglandPorton Down, UK; ^3^Cardiff School of Pharmacy and Pharmaceutical Sciences, Cardiff UniversityCardiff, UK

**Keywords:** *Bacillus anthracis*, decontamination, environmental, peracetic acid, bacterial spores, germination

## Abstract

Elimination of *Bacillus anthracis* spores from the environment is a difficult and costly process due in part to the toxicity of current sporicidal agents. For this reason we investigated the ability of the spore germinants L-alanine (100 mM) and inosine (5 mM) to reduce the concentration of peracetic acid (PAA) required to inactivate *B. anthracis* spores. While L-alanine significantly enhanced (*p* = 0.0085) the bactericidal activity of 500 ppm PAA the same was not true for inosine suggesting some form of negative interaction. In contrast the germinant combination proved most effective at 100 ppm PAA (*p* = 0.0009). To determine if we could achieve similar results in soil we treated soil collected from the burial site of an anthrax infected animal which had been supplemented with spores of the Sterne strain of *B. anthracis* to increase the level of contamination to 10^4^ spores/g. Treatment with germinants followed 1 h later by 5000 ppm PAA eliminated all of the spores. In contrast direct treatment of the animal burial site using this approach delivered using a back pack sprayer had no detectable effect on the level of *B. anthracis* contamination or on total culturable bacterial numbers over the course of the experiment. It did trigger a significant, but temporary, reduction (*p* < 0.0001) in the total spore count suggesting that germination had been triggered under real world conditions. In conclusion, we have shown that the application of germinants increase the sensitivity of bacterial spores to PAA. While the results of the single field trial were inconclusive, the study highlighted the potential of this approach and the challenges faced when attempting to perform real world studies on *B. anthracis* spores contaminated sites.

## Introduction

Anthrax, caused by the bacterium *Bacillus anthracis*, is primarily a disease of animals which can infect humans through contact with infected animals and their products, or as a consequence of bioterrorism (Baillie, 2009). The ability of the pathogen to form spores which are resistant to a range of environmental insults, but retain the ability to infect susceptible individuals, has resulted in the pathogen being employed as a biological weapon as was demonstrated during the 2001 US mail attacks (Atlas, 2002).

In addition to the tragic loss of life, these attacks highlighted the challenge faced by those tasked with dealing with the aftermath of a spore attack. The very properties which make *B. anthracis* an effective weapon, the ability to form chemically resistant spores, also makes them extremely difficult to remove from a contaminated environment without recourse to toxic chemicals (Manchee et al., 1994; Schmitt and Zacchia, 2012).

While the impact of toxic chemicals such as formaldehyde and chlorine dioxide can be controlled and mitigated within confined areas such office buildings, the same cannot be said for large urban spaces such as major transport hubs and city streets. The wide-scale use of large quantities of toxic compounds could have a major impact on the environment and on socio-economic activity (Franco and Bouri, 2010; Baillie and Theriault, 2013). The successful decontamination of 4 hectares of *B. anthracis* spore contaminated land on Gruinard Island, an uninhabited island located off the coast of Scotland which was contaminated by the military during World War 2 required the application of 2 million liters of 5% formaldehyde in sea water (Manchee et al., 1994). The release of a similar volume of toxic chemical in a major urban center could cause considerable environmental damage.

Thus there is a need to develop more benign decontamination approaches which retain the ability to inactivate the pathogen but cause less damage to the local flora and fauna if released in large quantities. Peracetic acid (PAA) is an oxidizing agent which breaks down into acetic acid, hydrogen peroxide, oxygen, and water and is currently being explored as a less damaging, more environmentally friendly alternative for large scale *B. anthracis* spore decontaminant. Its powerful antimicrobial action at low temperatures combined with the absence of toxic or mutagenic by-products in the presence of organic material has resulted in its wide spread use in applications ranging from waste water treatment and agricultural decontamination to the food-processing and beverage industries were it is considered to be ideal for clean-in-place systems (Bruins and Dyer, 1995; Kitis, 2004).

The ability of PAA it inactivate *B. anthracis* spores has been reported (Hussaini and Ruby, 1976; Hilgren et al., 2007; Majcher et al., 2008; Wood et al., 2013). In laboratory studies the agent has been shown to achieve between 4.78 and 6 log_10_ reduction in spores numbers at concentrations ranging from 3000 to 45000 ppm (0.3–4.5%). A small field trial on Gruinard Island confirmed the ability of 30,000 ppm (3%) to eliminate the pathogen from contaminated soil (Manchee et al., 1983).

In contrast a recent laboratory study using topsoil found that a mixture of hydrogen peroxide 27500 ppm (27.5%) and peroxyacetic acid 58000 pm (5.8%) in aqueous solution only achieved a 1 log reduction in *B. anthracis* spore numbers following repeated application (U. S. Environmental Protection Agency [EPA], 2010). These contrasting results probably reflect differences in the level of spore contamination and the nature of the test soils.

To enhance the efficacy of PAA against *B. anthracis* spores and thus further reduce any potential for environmental damage, we are exploring the possibility of making the spores convert to their chemically more sensitive vegetative forms. This is a process known as germination and in the laboratory can be stimulated by the presence of simple nutrients such as L-alanine and inosine (Barlass et al., 2002).

Recently it has been reported that the addition of L-alanine and inosine to a soil microcosm containing 10^7^ spores of the Sterne strain of *B. anthracis* resulted in a 6 log reduction in total bacterial numbers (both spores and vegetative bacteria) over a 2 weeks period at 22°C (Bishop, 2014). These results raise the intriguing possibility that by combining PAA with L-alanine and inosine it may be possible to potentiate the antibacterial activity of PAA against *B. anthracis* spores in soil and thus reduce the concentration of biocide required to inactivate the pathogen.

In this study we examined the ability of germinants to potentiate the activity of PAA against *B. anthracis* spores in the laboratory and in a small field trial on an anthrax spore contaminated animal burial site in North East Turkey where the disease in cattle is endemic and contaminated land represents an ongoing source of infection (Doganay and Metan, 2009).

## Materials and Methods

All reagents and chemicals were obtained from Fisher Scientific (Loughborough, UK) or Sigma–Aldrich (Dorset, UK) unless otherwise stated in the text. The *B. anthracis* Sterne culture used in this study was obtained from the Biological Defense Research Directorate of the US Navy.

### Production of *B. anthracis* Spores

A single colony of a 24 h culture of the Sterne strain of *B. anthracis* was emulsified into 9 mL of Brain Heart Infusion broth and 5 mL of this suspension was used to inoculate a Falcon vented flask (BD Falcon, vented cap) containing 200 mL of sterile growth medium at pH 6.7 (0.6% w/v nutrient broth, 1.2% w/v tryptone soya agar, 0.03% w/v manganese sulfate, 0.025% w/v sodium phosphate monobasic). A total of 10 flasks were inoculated per run. Each flask was incubated at 37°C for 10 days after which the spores were harvested and resuspended into 15 mL of distilled water. The suspension was then centrifuged at 3800 *g* for 15 min at 4°C, the supernatant was discarded and the pellet re-suspended in 20 mL of 70% (v/v) ethanol by agitating the tube horizontally for 60 min at 250 revolutions per minute (RPM). The supernatant was further centrifuged at 3800 *g* for 15 min at 4°C after which the pellet was re-suspended with 10 mL of water and heat treated at 65°C for 30 min in a circulating type water bath (Nüve, Turkey) to inactivate any vegetative organisms. Following a final centrifugation step as described above the spore pellet was re-suspended in 10 mL of sterile water at which stage viable counts and spore stain were performed to assess the concentration of spores in the final suspension.

### Neutraliser Efficacy Test

The vegetative form of *B. anthracis* is considerably more susceptible to biocide than the spore form. Following exposure to biocide it is important that a neutralization step is included to ensure that there is no residual agent which could target germinating bacteria and thus give a false impression of anti-sporicidal activity. Neutralizer efficacy was determined in accordance with the requirements of the EN 13697 method (BSI, 2001). Sodium thiosulphate solution (0.5% w/v Fisher Scientific, Loughborough, UK) was assessed for its ability to neutralize a solution containing 25000 ppm PAA which was made immediately prior to the experiment from a stock solution of PAA (32% in acetic acid; Sigma–Aldrich, Dorset, UK). One mL of *B. anthracis* spores (10^6^ spores/mL) was added to a 10 mL solution that contained 1 mL of biocide and 9 mL of neutraliser that had been pre-incubated for 5 min. This mixture was then incubated at the required temperature (4, 20, and 37°C) for 120 min in the presence (dirty) or absence (clean) of organic load (0.3% w/v Bovine Serum Albumin; Sigma–Aldrich, Dorset, UK). Following incubation, suspensions were diluted 1:10 in TSC and viable counts performed into duplicate Tryptone soy agar pour plates (TSA; Oxoid, Basingstoke, UK). All experiments were performed in triplicate and negative controls using sterile distilled water (SDW) were also performed. The reduction factor (the effectiveness of the neutraliser) is calculated as follows; Log_10_ (CONTROL) – Log_10_ (BIOCIDE TREATED). Reduction factors must be <0.3 Log_10_ to consider the neutralization step valid.

### Assessment of PAA Activity Against *B. anthracis* Sterne Spores

The bactericidal activity of PAA (25000, 10000, 5000, and 1000 ppm) against spores of *B. anthracis* Sterne at different temperatures (4°, 20°, and 37°C) and in the presence and absence of an organic load (0.3% w/v BSA) was assessed using the British standard EN 1276 suspension test (BSI, 2002). In brief, one mL *B. anthracis* Sterne spores (containing approximately 10^8^ spores/mL) was added to 9 mL PAA. All working solutions of PAA were made from the stock solution immediately prior to use. This was then incubated at the desired conditions for 30, 60, 120 min prior to neutralization in 0.5% w/v sodium thiosulfate and left at room temperature for 5 min. Neutralized samples were then serially diluted 1:10 and total viable count performed on duplicate Tryptone soy agar (TSA; Oxoid, UK) plates. Plates were then incubated at 37°C for 24 h.

### Assessment of PAA Activity Against *B. anthracis* Sterne Spores with and without Spore Germinants

The sporicidal activity of germinants (100 mM L-alanine and 5 mM inosine) alone and in combination with PAA (1000, 500, and 100 ppm) was assessed at 20°C in the absence of organic load for a contact time of 30 min using the method described above.

### Collection and Characterisation of *B. anthracis* Spores Contaminated Soil from an Animal Contaminated Sites in Turkey

Soil was collected from two sites which had yield *B. anthracis* spores on previous occasions (Buyuk et al., 2015). Fifty gram samples from the top 2 cm of the soil were removed using a metal trowel from each of five randomly selected sectors of a 2 by 2 m grid constructed with metal pegs and string which had been overlaid over the center of the contaminated site. Samples were then transferred to clean 500 ml sample pots and returned to the laboratory for processing.

Upon arrival at the laboratory the soil was mixed with 100 ml of SDW for 1 min by hand and left to settle for 10 min. At the end of this period 1 ml of supernatant was serially diluted 1 in 10 dilutions in SDW and 150 μL spread over the surface of duplicate Columbia Sheep’s Blood agar plates (Oxoid, UK). To determine total viable content plates were incubated at 37°C for 24 h. To determine the spore count, 1 ml of supernatant was heat shocked at 70°C for 40 min in a static water bath and then enumerated as above. The identity of suspected *B. anthracis* colonies was confirmed on the basis of morphology, capsule expression, Gamma phage, and penicillin susceptibility (Anthrax in Humans and Animals, World Health Organization [WHO], 2008). The lower limit of detection of this method was 13.2 cfu/g of soil.

### Assessment of the Activity of the PAA/Germinant Mixture Against *B. anthracis* Spores Artificially Introduced into Soil Collected from Test Site B in the Laboratory

Due to the low level of natural contamination, the *B. anthracis* spore load of soil samples collected from the contaminated site was artificially increased to 10^4^ spores/g of soil by the addition of spores of the Sterne strain of *B. anthracis*. Biocide activity in the presence and absence of germinants was determined as follows: Soil (5 g) was mixed with 5 mL of 5000 ppm PAA, and applied with or without a germinant mixture comprising 100 mM L-alanine and 5 mM inosine. The effect of adding the germinant first followed by the biocidal 1 h later was also assessed. Samples were incubated at room temperature for the required PAA contact time (1, 2, and 24 h). One gram aliquots post PAA exposure were neutralized with 2 mL 0.5% (w/v) sodium thiosulfate. Neutralized samples were then serially diluted in SDW and 10 μL spread over the surface of duplicate Columbia Sheep’s Blood agar plates (Oxoid, UK) to determine total spore and vegetative contents. Each assay was repeated in triplicate. The lower limit of detection of this method was 200 cfu/g.

### Assessment of the Activity of the PAA/Germinant Mixture Against *B. anthracis* Spores at Test Site B

A 2 m × 2 m area at test site B was treated with 16 L of germinant (100 mM L-alanine and 5 mM inosine) followed 1 h later by 16 L of PAA (5000 ppm). Each agent was applied using a Micropak KS16 Knapsack backpack Sprayer (Maxwell Amenity Ltd, Telford, UK).

To determine the effect of treatment on *B. anthracis* spores numbers, soil samples were collected prior to decontamination immediately before, and at 1 and 24 h post biocide application. On each occasion 5 g × 50 g soil samples comprising the top 2 cm of topsoil were collected using a 2 m × 2 m sampling grid. Immediately following collection, each 50 g sample was mixed with 100 mL of 0.5% (w/v) sodium thiosulfate to neutralize residual biocide activity. Total vegetative and total spore content was then determined as described above, however, for this experiment 150 μl was spread over the surface of duplicate plates. The lower limit of detection of this method was 13.2 cfu/g of soil.

### Degradation of PAA by Test Soil

The breakdown of liquid PAA was assessed in the laboratory following application to soil samples taken from site A. To a 5 g sample of soil, 5 mL of biocide (10000, 5000, and 1000 ppm) was added. Samples were then shaken and the level of residual PAA determined using Quantofix peroxide test strips (Sigma–Aldrich, Dorset, UK) at different times post application (1, 2, and 24 h). Soil samples collected from test site B 1 and 24 h following application of PAA were also tested for the presence of PAA using test strips to determine the degradation of the biocide under real world conditions.

### Compliance with Local Regulations

All studies in which biocides were used to treat *B. anthracis* spore contaminated sites received prior approval from the Turkish Department of the Environment and Food, Agriculture and Livestock. All studies which involved the handling of fully virulent strain of *B. anthracis* were undertaken in the bio-containment facilities at Kafkas University.

### Data Analysis

Where appropriate, data was analyzed using a Student’s *t-*test and graphpad software (http://graphpad.com/quickcalcs/ttest1.cfm).

## Results

### Confirmation of Neutraliser Efficacy

To determine the biocidal effect of the treatment during the contact period we assessed the ability of 0.5% (w/v) Sodium thiosulphate to neutralize the biocidal activity of 25000 ppm PAA (the highest concentration of agent used in these studies). As can be seen in **Table 1** the difference between treated and untreated samples across all of the temperatures tested and in the presence and absence of organic material was less than 0.3 log_10_ indicating that the treatment had been successful.

**Table 1:** Neutraliser efficacy test.

		Incubation temperature (°C)					
		4		20		37	
		Clean	Dirty	Clean	Dirty	Clean	Dirty
Sodium thiosulphate (0.5% w/v)	CFU	6.71 (0.02)	6.68 (0.05)	6.68 (0.01)	6.64 (0.02)	6.72 (0.02)	6.65 (0.06)
Peracetic acid (25000 ppm)	CFU	6.58 (0.05)	6.54 (0.08)	6.57 (0.05)	6.60 (0.05)	6.69 (0.05)	6.50 (0.04)
	RF	0.13 (0.05)	0.14 (0.08)	0.11 (0.05)	0.04 (0.05)	0.04 (0.05)	0.15 (0.04)

*CFU, Log_10_ CFU; RF, reduction factor. Data is expressed as log_10_. Data are the mean of three experiments (±SD). Clean = No Bovine Serum Albumin present, Dirty = 0.3% w/v Bovine Serum Albumin.*

### Assessment of Biocidal Activity in the Laboratory using a Suspension Assay

The ability of liquid PAA to inactivate spores of the Sterne strain of *B. anthracis* in suspension across a range of test conditions was determined using the British Standard EN 1276 suspension test (BSI, 2002). According to this standard, a biocide is considered to have a “bactericidal effect” if it achieves a reduction of ≥5 log_10_ in viable bacteria. All concentrations of PAA (25000–1000 ppm) achieved a ≥5 log_10_ reduction in spores numbers within 30 min for all conditions tested in the presence and absence of organic load (**Table 2**).

**Table 2:** The sporicidal activity of PAA for *Bacillus anthracis* spores using a suspension test assay format.

Suspension test					
			**Reduction factors (Log_10_)**		
**Concentration (ppm)**	**Temperature (°C)**	**Clean or dirty condition**	**Contact time (min)**		
			**30**	**60**	**120**
25000	4	Clean	>5	>5	>5
		Dirty	>5	>5	>5
	20	Clean	>5	>5	>5
		Dirty	>5	>5	>5
	37	Clean	>5	>5	>5
		Dirty	>5	>5	>5
10000	4	Clean	>5	>5	>5
		Dirty	>5	>5	>5
	20	Clean	>5	>5	>5
		Dirty	>5	>5	>5
	37	Clean	>5	>5	>5
		Dirty	>5	>5	>5
5000	4	Clean	>5	>5	>5
		Dirty	>5	>5	>5
	20	Clean	>5	>5	>5
		Dirty	>5	>5	>5
	37	Clean	>5	>5	>5
		Dirty	>5	>5	>5
1000	4	Clean	>5	>5	>5
		Dirty	>5	>5	>5
	20	Clean	>5	>5	>5
		Dirty	>5	>5	>5
	37	Clean	>5	>5	>5
		Dirty	>5	>5	>5

**FIGURE 1:**
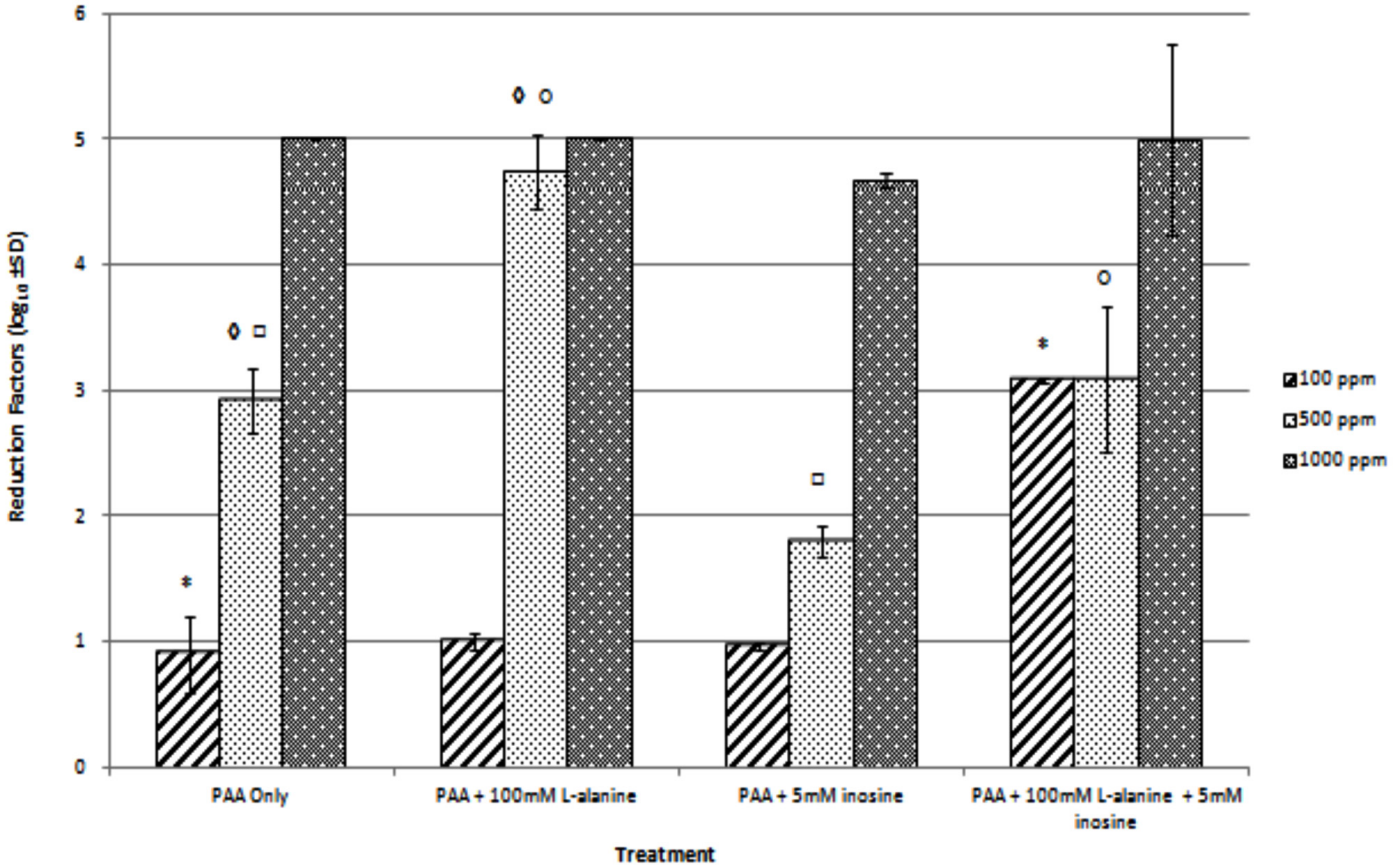
**The effect of germinants, along and in combination on the biocidal efficacy of PAA against *Bacillus anthracis* Sterne spores was assessed at 20°C for a contact time of 30 min.** Data are the mean of three replicates ±SD. ^∗^ ♢ □ ○; significant differences (*p* = 0.0009, 0.0085, 0.0024, 0.0361, respectively) between results as determined using a Student’s *t*-test.

To determine if we could further decrease the concentration of PAA required to inactivate *B. anthracis* spores, we examined the effect of the co-delivery of spore germinants alone or in combination with different concentrations of the biocide. As can be seen from **Figure 1**, exposure to 1000 ppm of PAA alone resulted in a 5 log reduction in spore viability confirming the earlier data. Reducing the concentration to 500 and 100 ppm achieved 2.9 and 0.9 log reductions in spore numbers over the same time period suggesting a concentration dependent effect. The addition of the germinant alanine had no effect on biocidal activity in the presence of 1000 ppm PAA but significantly increased killing (*p* = 0.0085) in the presence of 500 ppm. In contrast to the potentiating effect of alanine on biocide efficacy the presence of inosine significantly inhibited the ability of 500 ppm PAA to inactivate spores (*p* = 0.0024). A similar inhibitory effect was found when alanine and inosine were both present (*p* = 0.0361). Interestingly this effect was not seen when the concentration of PAA was reduced to 100 ppm suggesting a concentration dependent effect. Indeed the combination of alanine and inosine with 100 ppm PAA had a significant effect (*p* = 0.0009) on bactericidal activity when compared to 100 ppm PAA alone (**Figure 1**). While the level of inactivation achieved by this formulation would not be considered bactericidal as defined by the British Standard EN 1276 it does demonstrate the ability of germinants to potentiate the activity of PAA against *B. anthracis* spores.

### Characterisation of Potential Test Sites in Turkey

While laboratory studies provide useful information, they are only a reflection of what is likely to occur in the real world. For this reason two test sites were selected: site A which was a site at which an infected cow had been slaughtered in 2010 and site B where three cows that had died of anthrax and had been buried in 2006. The mean level of *B. anthracis* spore contamination (samples of the top 2 cm of soil randomly collected from five different points of a 2 m × 2 m grid) for site A was 11.8 spores/gram (60% samples culture positive) while for site B the count was 1320 spores/gram (20% samples culture positive).

### Assessment of the Activity of the PAA/Germinant Mixture Against ***B. anthracis*** Spores Artificially Introduced into Soil Collected from Test Site B in the Laboratory

**FIGURE 2:**
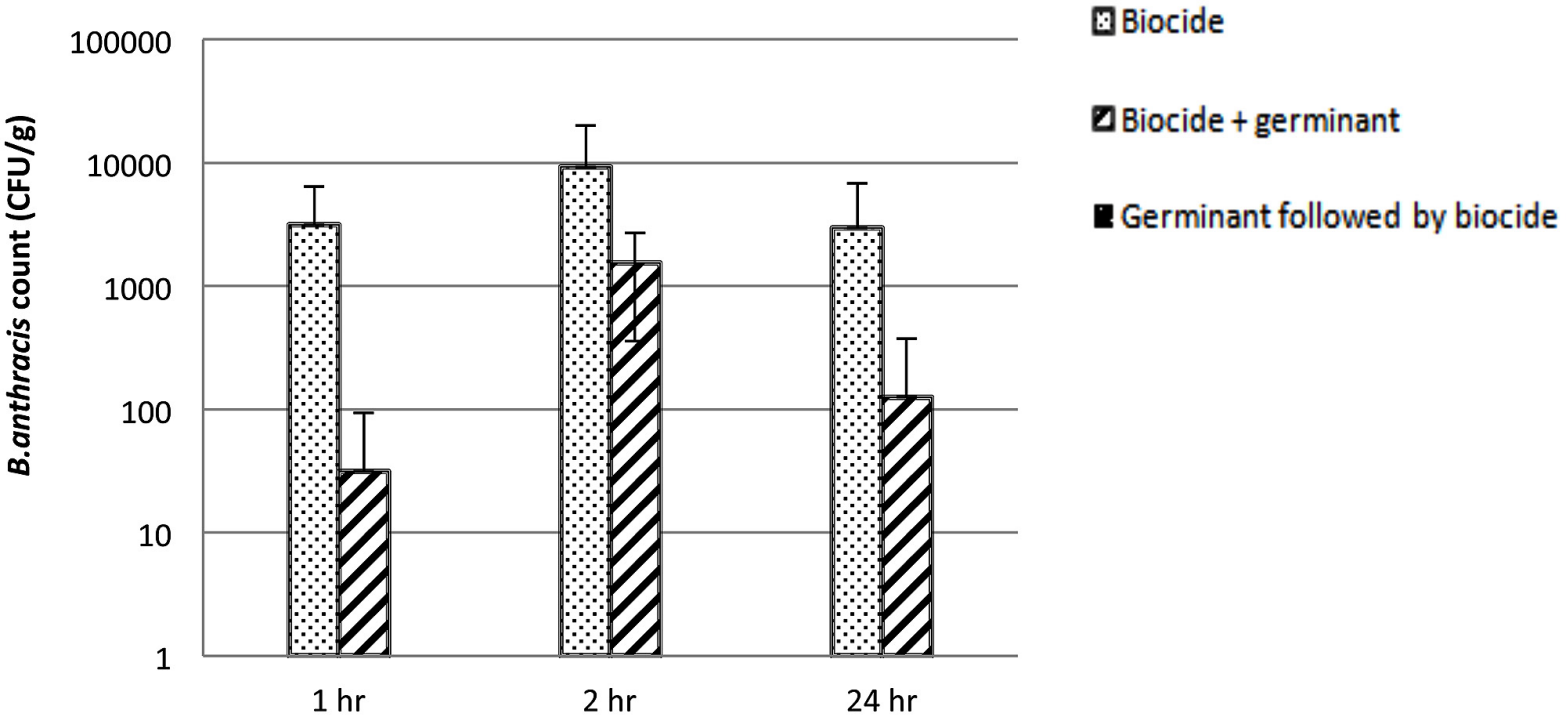
**The effect of germinants on the biocidal activity of 5000 ppm PAA against spores of the Sterne strain of *B. anthracis* suspended in soil collected from test site B.** The bar indicates the number of *B. anthracis* remaining following treatment. Data are the mean of two replicates ±SD. The lower limit of detection was 200 cfu/g of soil per assay.

To determine the optimum delivery strategy for the germinants and the biocide we supplemented soil collected from test site B in the laboratory with spores of the Sterne strain of *B. anthracis* to achieve a final concentration of 5.6 × 10^4^ spore/gram. We then investigated the effect of treating the soil as follows; 5000 ppm PAA alone, 5000 ppm PAA co-delivered with germinants and germinants followed 1 h later by 5000 ppm PAA. As can be seen from **Figure 2** the degree of *B. anthracis* inactivation varied between different treatment formulations.

While treatment with PAA alone caused a reduction in spore numbers it did not eliminate all of the *B. anthracis* as was the case for the contaminated soil treated with germinants followed by biocide. Interestingly we saw no significant difference in the reduction in *B. anthracis* numbers when the results for the PAA and PAA + germinants were compared across all of the time points.

### Biocide Treatment of *B. anthracis* Spore Contaminated Test Site B

**FIGURE 3:**
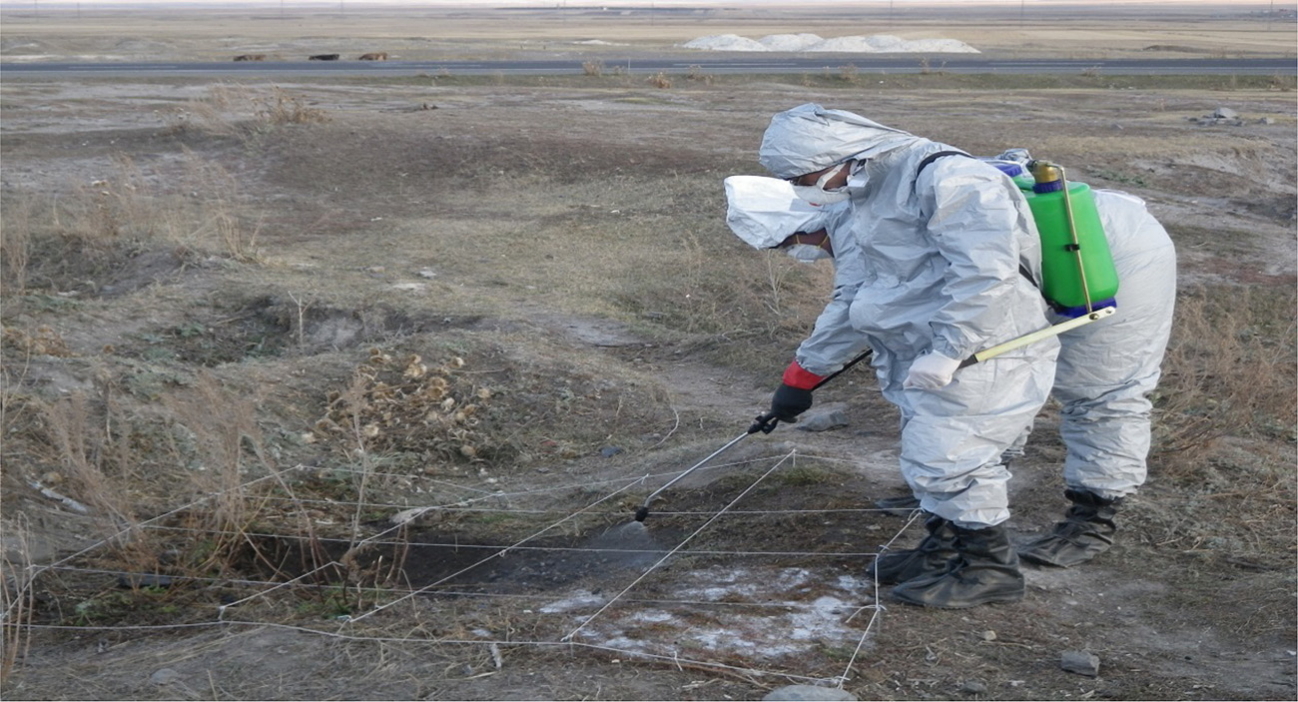
**Treatment of test site B with germinants and biocide delivered using a Micropak KS16 backpack sprayer**.

Even thought the level of natural *B. anthracis* spore contamination at test site B was relatively low we wanted to take advantage of this unique opportunity to generate real world data. Thus test site B was treated with a regimen comprising a mixture of germinants followed 1 h later by PAA at a concentration of 5000 ppm which was the maximum concentration permitted by the Turkish authorities, delivered using a back pack sprayer (**Figure 3**). An advantage of this system is the ability to specifically target the delivery of the treatment and, as a consequence, each m^2^ of test site received 4 L of germinant mixture followed by 4 L of biocide. The effect of this treatment on bacterial numbers is shown in **Table 3**. The low level of natural *B. anthracis* spores contamination coupled with its uneven distribution across the test site meant that we were unable to show any significant effect of treatment on the level of the pathogen. For this reason we also determined the effect of treatment on the number of culturable bacteria we were able to recover. As can be seen over the course of the experiment there was no significant change in total bacterial numbers although we did see a significant reduction (*p* < 0.0001) in the total spore count following the addition of germinants suggesting that germination had indeed been triggered. This was a temporary effect as spores numbers returned to close to the starting level 1 h later.

**Table 3:** Total vegetative and spore content of environmental samples treated with 5000 ppm PAA and germinants (100 mM L-alanine and 5 mM inosine) using the MicroPak KS16 backpack sprayer.

Test site and delivery method	Treatment	Total culturable bacterial content -log_10_ CFU/g		*B. anthracis* content -log_10_ CFU/g	
		Vegetative	Spores	Vegetative	Spores
Site B MicroPak KS16	Untreated	5.98 (0.69)	5.09 (0.23)^∗^	0.60 (1.34)	1.03 (1.42)
	1 h post germinant	6.02 (0.48)	3.68 (0.22)^∗^	-^a^	-^a^
	1 h post biocide	5.20 (0.74)	4.83 (0.23)	0.72 (1.61)	-^a^
	24 h post biocide	5.74 (0.70)	4.83 (0.18)	0.40 (0.89)	0.40 (0.89)

Data shown are the mean of five replicates collected at the same time point (±SD). The lower limit of detection of this method was 13.2 cfu/g of soil. ^a^B. anthracis not detected, *p < 0.0001.

### The Breakdown of PAA in Soil from Contaminated Sites

An area of considerable concern to the Turkish authorities was the possibility that the biocide might cause collateral damage to environment. Indeed, PAA was selected for these studies because it breaks down into environmentally harmless compounds. Laboratory studies confirmed using test soil from site A that PAA at 5000 ppm could not be detected 2 h following application (**Table 4**).

**Table 4:** Breakdown of liquid PAA following application to soil obtained from site A.

Biocide Concentration (ppm)	Time post PAA addition (h)			
	0	1	2	24
10000	2000	1000	<500	0
5000	2000	<1000	0	0
1000	1000	<500	0	0

Immediately following application of PAA to the test soil, which was covered in places by grass, we observed what appeared to be the release of bubbles of gas from the treated surfaces. Testing of the soil revealed that the level of PAA had reduced to 2000 ppm. Given that oxidizing agents such as PAA are known to break down into water, oxygen and low levels of acetic acid this observation suggests that factors present in the environment rapidly reduce the biocidal activity of the agent.

Following the field studies the level of PAA at site B 1 h post biocide treatment was less than 500 ppm while at 24 h the level was below 5 ppm confirming that the biocide was rapidly degraded as expected.

## Discussion

The feasibility of reducing the number of *B. anthracis* spores in a contaminated building by the application of toxic chemicals such as formaldehyde and chlorine dioxide has been demonstrated (Schmitt and Zacchia, 2012). Unfortunately these processes are time consuming and would be extremely expensive if employed to decontaminate a large urban space such as a major transport hub (Manchee et al., 1994). In the aftermath of the 2001 US anthrax attacks, some of the affected sites were closed for up to 2 years and the associated decontamination costs were in the order of $320 million (Schmitt and Zacchia, 2012).

The prolonged closure of a major transport hub in an urban center as a consequence of an Anthrax attack would have a major impact on economic activity. For this reason, there is a need to develop environmentally friendly decontamination approaches which could be rapidly deployed to treat contaminated urban environments. Compared to a number of other biocides, PAA is considered to be environmentally friendly and as a consequence is widely used as an environmental decontaminant (Bruins and Dyer, 1995; Kitis, 2004). Its activity against *B. anthracis* spores has lead to it being recommended as an agent with which to treat spore contaminated agricultural land (Hussaini and Ruby, 1976).

While the level of sporicidal activity observed in our laboratory studies was of a similar order of magnitude to that reported by others (Hussaini and Ruby, 1976; Hilgren et al., 2007; Majcher et al., 2008; Wood et al., 2013), we were able to enhance low level biocidal activity by the co-delivery of the germinants L-alanine and inosine. These compounds have been shown to be amongst the most efficient triggers of *B. anthracis* germination converting the relatively biocide resistant spore into its considerably more sensitive vegetative form (Gould et al., 1968; Ireland and Hanna, 2002; Moir, 2006; Omotade et al., 2013). While L-alanine significantly potentiated biocidal activity the same was not true for inosine which appeared to inhibited sporicidal activity in the presence of 500 ppm PAA. This inhibition is probably a concentration dependent effect as when both germinants were co-delivered with PAA at 100 ppm they stimulated a significant increase in biocidal activity. We have observed a similar negative interaction between chlorine based biocides and L-alanine (Williams and Baillie, 2009).

Given that we are attempting to develop an environmental decontaminant we next determined the ability of these germinants in combination with PAA to reduce the level of *B. anthracis* in artificially contaminated soil collected from an animal burial site. Treating the soil with germinants followed 1 h later with 5000 ppm PAA, the maximum concentration of biocide permitted by the Turkish authorities for use on contaminated land, was more effective at reducing bacterial numbers than delivering them at the same time. This result adds further support to the hypothesis that there is a concentration dependent negative interaction between the germinants and PAA.

While laboratory based studies provide useful information, they are only an approximation of the real world. For this reason we treated site B with germinants followed 1 h later by 5000 ppm PAA delivered by backpack sprayer. Due to the low numbers of *B. anthracis* recovered from the site we also determined the effect of treatment on total culturable bacterial numbers.

While treatment of site B had no significant effect on the level of *B. anthracis* contamination or on the total culturable bacterial numbers, we did see a significant reduction in the total spore count following the addition of germinants suggesting that germination had been triggered under real world conditions. This was a temporary effect as spores numbers returned to near to the starting level 1 h after the addition of PAA.

This reformation of spores could be a reflection of the newly sporulated bacteria exploiting the germinants and acetic acid, a non-toxic breakdown product of PAA, as nutrients to support bacterial replication and subsequent spore formation (Kitis, 2004). Alternatively the bacteria may be undergoing a process known as microcycle sporogenesis in which newly germinated spores convert back to dormant spores without the need for cell division (Vinter and Slepecky, 1965; MacKechnie and Hanson, 1968). These results differ from those reported by Bishop who saw increased germination over time in the presence of alanine and inosine and highlight the importance of taking local soil factors into account when attempting to develop decontamination strategies (Hugh-Jones and Blackburn, 2009).

While we were able to stimulate spore germination in contaminated soil, and to demonstrate enhanced PAA mediates *B. anthracis* spore inactivation in soil microcosms, we were unable to detect a similar level of inactivation in the field. There are a number of possible reasons such as the low level of natural contamination, the uneven distribution of the *B. anthracis* spores across the test site and the presence of factors in soil such as transition metals which catalyze the breakdown of oxidizing biocides via the Fenton reaction (Huang et al., 2001). Indeed the presence of such metals may explain the rapid breakdown of PAA which was seen in this study. It may be possible to increase the efficacy of this approach by using L-alanine alone, by increasing the gap between germinant delivery and biocide treatment to give the spores more time to germinate and by increasing the concentration and volume of PAA.

It should also be remembered that these results are from a single field trial which due to the nature of the agent took over 2 years to negotiate. While it was our intension to perform additional field trials operational issues and a change in the weather prevented us from completing them. Thus further field trails a required to determine to the true to utility of this approach.

## Conclusion

We have shown that the application of germinants increase the sensitivity of bacterial spores to PAA. While the results of the single field trial were inconclusive, the study has highlighted the potential of this approach and the challenges faced when attempting to perform real world studies on *B. anthracis* spores contaminated sites.

## Author Contributions

All authors listed, have made substantial, direct and intellectual contribution to the work, and approved it for publication.

## Conflict of Interest Statement

The authors declare that the research was conducted in the absence of any commercial or financial relationships that could be construed as a potential conflict of interest.
